# Do current maternal health staffing and bed occupancy benchmarks work in practice? Results from a simulation model

**DOI:** 10.1136/bmjph-2023-000212

**Published:** 2024-01-19

**Authors:** Rebecca F Baggaley, Giorgia Gon, Said Mohammed Ali, Salma Abdi Mahmoud, Farhat Jowhar, Carolin Vegvari

**Affiliations:** 1 Department of Population Health Sciences, University of Leicester, Leicester, UK; 2 Department of Infectious Disease Epidemiology, Imperial College London, London, UK; 3 Department of Infectious Disease Epidemiology and International Health, London School of Hygiene & Tropical Medicine, London, UK; 4 Department of Public Health Laboratory-Ivo de Carneri (PHL-IdC), Public Health Laboratory, Chake Chake, Tanzania, United Republic of; 5 The State University of Zanzibar, Zanzibar, Tanzania, United Republic of; 6 Integrated Reproductive and Child Health Program, Zanzibar Ministry of Health, Zanzibar, Tanzania, United Republic of; 7 Oriole Global Health, London, UK

**Keywords:** public health, epidemiology, community health, statistics and numerical data

## Abstract

**Introduction:**

The WHO has issued the global target of reducing maternal mortality rates by two-thirds of 2010 baseline levels by 2030. In low-income settings, high birth rates and a relative lack of medical resources mean that an efficient use of resources and skilled staff is important in ensuring quality of intrapartum and postpartum care.

**Methods:**

We use a stochastic, individual-based model to explore whether WHO resourcing benchmarks are sufficient to ensure consistent quality of care. We simulate all deliveries occurring in a region over a year, with date and time of presentation of each woman delivering at a facility assigned at random. Each woman stays in the delivery room for an assigned duration before her delivery, then moves to the maternity ward, followed by discharge. We explore the potential impact of seasonality of births on our findings and then apply the model to a real-world setting using 2014 data from Emergency Obstetric Care (EmOC) facilities in Zanzibar, United Republic of Tanzania.

**Results:**

We find that small EmOCs are frequently empty, while larger EmOCs are at risk of temporarily falling below minimum recommended staff-to-patient ratios. Similarly for Zanzibar, capacity of EmOCs in terms of beds is rarely exceeded. Where over-capacity occurs, it is generally smaller, basic EmOCs (BEmOCs) that are affected. In contrast, capacity in terms of staffing (skilled birth attendants:women in labour ratio) is exceeded almost 50% of the time in larger Comprehensive EmOCs (CEmOCs).

**Conclusions:**

Our findings suggest that increasing staffing levels of CEmOCs while maintaining fewer small BEmOCs may improve quality of care (by increasing the staff-to-patient ratio for the most frequently used facilities), provided that timely access to EmOCs for all women can still be guaranteed. Alternatively, BEmOCs may need to be upgraded to ensure that women trust and choose these facilities for giving birth, thus relieving pressure on CEmOCs.

**WHAT IS ALREADY KNOWN ON THIS TOPIC**

**WHAT THIS STUDY ADDS**

- Our simulation model demonstrated that under the WHO benchmark scenario, small birth facilities are frequently empty, while larger facilities such as hospitals are at risk of temporary understaffing.

**HOW THIS STUDY MIGHT AFFECT RESEARCH, PRACTICE OR POLICY**

- Our findings suggest that increasing staffing levels of larger facilities while maintaining fewer smaller facilities may improve quality of care, provided that timely access to facilities can still be guaranteed for all women.

## Introduction

 Access to Emergency Obstetric Care (EmOC) is critical to reduce intrapartum deaths of mothers and new-borns caused by pregnancy complications. The WHO recommendations for ending preventable maternal mortality state that countries with a maternal mortality ratio<420 in 2010 should reduce maternal mortality by at least two-thirds by 2030.^1^ In low-income settings, access to EmOC may be limited by a shortage of medical resources, especially the number of trained staff. In combination with high birth rates, this means that an efficient use of resources and trained staff will be essential to ensure high-quality obstetric care.

The WHO has set resourcing benchmarks for Basic Emergency Obstetric Care Centres (BEmOCs) and Comprehensive Emergency Obstetric Care Centres (CEmOCs) (box 1).^2^ These guidelines aim to ensure that there are sufficient skilled birth attendants (SBAs) and beds in the delivery room. The guidelines set out minimum provision of SBAs and beds as averages per number of births (see summary of recommendations, box 1).^2^ However, women do not enter delivery rooms in averages: onset of labour is unpredictable. Rather, women entering delivery rooms can be considered as stochastic (ie, random) events which by chance may cluster in time. In individual facilities, this can lead to a higher number of women entering delivery rooms than expected some of the time and underutilised capacity at other times. Surges in women entering delivery rooms can be exacerbated in settings with marked birth seasonality which has been observed for some sub-Saharan African countries.^3^

Box 1 **Summary of benchmarks for supply-side needs to provide universal maternal and newborn care, as outlined in The World Health Report 2005 (chapter 5).^2^ (Recommendations preceding The World Health Report were described in the report as being one CEmOC and four BEmOC facilities per 500 000 population, equivalent to 3000 births per year.)**

**Arrangement 1:**

Team 1: 9–10 midwives/SBAs in hospital; 10–11 in BEmOCs.

**Arrangement 2 (more dispersed population):**

Five midwives/SBAs per facility (for 24 hours availability) plus emergency evacuation costs.

**Arrangement 3 (large, sparsely populated districts):**

Individual midwives in villages.

Benchmarks for filling the supply gap to scale up first-level and back-up maternal and newborn care in 75 countries (from the current 43% to 73% coverage by 2015 and full coverage in 2030).

The 2005 World Health Report^2^ recommends the following minimum facility and staff provisions for first-level maternal and newborn care for all mothers and newborns:

- One birthing centre per 1750 births.
- One midwife or other professional with midwifery skills per 175 births.

Moreover, the 2005 World Health Report recommends the following hospital provisions for back-up maternal and newborn care for at least 7% of mothers and 9%–15% of newborns:

- One hospital per 120 000 inhabitants.

*An average district in southeast Asia is acknowledged to be much smaller than this.

BEmOCs, Basic Emergency Obstetric Care Centres; CEmOCs, Comprehensive Emergency Obstetric Care Centres; SBAs, skilled birth attendants.

Over-capacity delivery rooms and maternity wards risk insufficient staff-to-patient ratios to maintain acceptable standards of care for women during and after birth or women being discharged too early to free up capacity in terms of beds and staff as demonstrated in low-income settings,^4^ although the situation in high-income settings is less clear.^5 6^ Conversely, overprovisioning of staff and beds may represent an inefficient use of resources, especially in resource-poor settings. According to WHO benchmarks, SBAs ‘can easily assist at least 175 births per year’.^2^ In smaller facilities, SBAs may see too few births and especially too few complicated births to maintain training. In contrast, in larger facilities, SBAs may have to attend a substantially higher number of births and thus risk being overworked and decreasing quality of care. There is therefore a trade-off between resilience (avoiding risk to mothers and new-borns due to dangerously high SBA workload levels) and efficient use of limited resources.

In this study, we present a stochastic, individual-based model of women entering and leaving delivery rooms and maternity wards that can help public health planners to identify the right balance between resilience and efficiency of EmOC provisions. Using such a model, we can assess and juxtapose the risks of falling below minimum recommended staff-to-patient ratios versus inefficiency, given predetermined levels of staffing and number of beds in different EmOC facilities. First, we present two generic scenarios for sub-Saharan Africa and compare the outcomes with WHO EmOC benchmarks.^2^ We then apply our model to EmOC data that were collected in 2014 in Zanzibar as a case study to inform necessary improvements and investment requirements into hospital infrastructure and staffing. Zanzibar has since built new hospitals and upgraded existing healthcare facilities, including facilities with EmOC functionality. Our analysis based on the 2014 data set should therefore be considered as a historic case study, rather than representative of the current situation in Zanzibar. We decided to use this data set for our analysis because it is still representative of the situation of perinatal care in many low-income settings and because an updated data set including the new EmOC facilities is not yet available, as most of them have only opened recently. For both the WHO benchmark analysis and the Zanzibar analysis, we additionally explore the potential impact of seasonality in births.

## Methods

### Model description

We use a stochastic, individual-based model to simulate all women entering and leaving delivery rooms and maternity wards over a period of 1 year (see online supplemental appendix 1 figure S1 for schematic diagram). The number of women modelled depends on the scenario (for the WHO benchmark scenario, it is the WHO estimate of numbers of births in a year in a ‘typical district’; for the Zanzibar scenario, it is informed by the data). Each delivery is modelled individually as a stochastic event. Multiple births are counted as one delivery event. The date and time of presentation of each woman delivering at a facility are assigned at random. The woman stays in the delivery room of the facility for an assigned duration before moving to the maternity ward after delivering her baby. After a second assigned duration, each woman leaves the maternity ward and is discharged from the facility. We assume that all women deliver in facilities and that there is no provision for referral between facilities which is representative for many resource-poor settings where referral between facilities can be difficult.

Online supplemental appendix S1 summarises all model inputs; 15% of deliveries are defined as complicated,^2 7^ assigned at random. Length of stay (LOS) in the delivery room is determined by complication status. Only limited data on the LOS in delivery rooms in resource-poor settings in sub-Saharan Africa are available. We fitted a gamma distribution to data on LOS in delivery rooms from Nigeria^8^ (see online supplemental appendix 1 for details), then censored to be a minimum of 2 hours. LOS in the maternity ward is also determined by complication status, and the distribution of LOS in the maternity ward has been found to be highly skewed.^9^ The LOS in the maternity ward for each woman is drawn randomly from a gamma distribution fitted to DHS data from sub-Saharan Africa^9^ (WHO benchmark analysis) or The United Republic of Tanzania (hereafter referred to as Tanzania) (Zanzibar case study).^10^ As women are not discharged from maternity wards at night, all discharge times falling between 8 pm and 8 am are delayed until 8 am the following morning.

The model outputs the number of women in delivery rooms and maternity wards of each facility at set time points. For each scenario and each facility, the number of women in the delivery rooms of the facility at 4 am, 12 pm and 6 pm each day is determined from the time when each woman enters the delivery room and her assigned LOS. The 4 am, 12 pm and 6 pm snapshots represent points in time mid-shift for SBAs in the EmOC facilities (shifts in Zanzibar start at 6 am, 2 pm and 8/9 pm), including one snapshot during the long, overnight shift. After delivery, each woman is transferred to the maternity ward for recovery. Maternity ward occupancy is assimilated in the same way as delivery room occupancy, with number of women in the maternity ward of each facility calculated each day at 4 am, 12 pm and 6 pm. We generated hourly snapshots of delivery room occupancy by comparing occupancy levels (number of women in delivery and maternity wards) with the number of beds available, to calculate the per cent of time when the capacity of each ward in each facility is exceeded. Similarly, we calculated the per cent of time when there are more women than SBAs in the delivery room so that one-to-one care, as recommended in many high-income countries,^11 12^ is not possible. We also calculated the per cent of time when delivery rooms in each facility are empty and the number of births per SBA per year in each facility. In order to assess the impact of stochasticity, we repeated each of the analyses described above 1000 times.

The model was implemented and simulations were run in R V.4.0.3.^13^ Model and analysis code are available at: https://github.com/kl3mn9/maternity-model.git.

### WHO benchmark analysis

The model represents the assumptions made by the WHO benchmarks^2^ for a typical sub-Saharan African district with around 120 000 inhabitants; assuming a birth rate of 30 per 1000 inhabitants, there are 3600 mothers giving birth per year (box 1). Each of the 3600 pregnant women in the model is assigned a health facility for delivery.

We consider the following two scenarios for size and distribution of maternity facilities in such a district:

Scenario A: one CEmOC facility staffed by 10 SBAs and two BemOC facilities each staffed by five SBAs.

Scenario B: one CEmOC facility staffed by 10 SBAs and five BEmOC facilities staffed by two SBAs each.

These scenarios broadly represent recommendations in the WHO 2005 report (see box 1).^2^ Data from sub-Saharan Africa suggest that women prefer to give birth in CEmOC facilities and that in resource-poor settings, it can be difficult to predict complications during birth.^14^ Therefore, we assume that the probability of births happening in CEmOCs is 50% regardless of complication status. The remaining births happen in BEmOCs with equal probabilities for each BEmoC facility.

For scenario B, where each BEmOC is only staffed by two SBAs (ie, only two shifts per day can be covered), we calculated how many women on average present at night when the delivery room is unstaffed. We assume that delivery rooms are staffed 06.00–14.00 (first 8 hour shift) and 14.00–22.00 (second 8 hour shift).

In this analysis, we chose to focus on delivery room occupancy rather than maternity ward occupancy because of the acute needs of women while in the delivery room, where understaffing and overoccupancy are likely to have the most important consequences.

### WHO benchmark analysis with seasonality

To investigate the potential impact of birth seasonality on utilisation and risk of exceeding capacity of EmOC facilities, we repeated the WHO benchmark analysis for a hypothetical setting with marked birth seasonality. We weighted the probability of a birth happening in a given month. Weights were derived from data on birth seasonality in Sierra Leone, the country with the greatest amplitude of birth seasonality presented in a study by Dorélien.^3^ We extracted data on the average monthly amplitude in births for Sierra Leone from figure 4B in online supplemental appendix B of Dorélien^3^ using Datathief.^15^ We estimated the probability of giving birth during each month of the year by dividing each monthly birth rate from the data by the total annual birth rate. The date and time of presentation of each woman delivering at a facility were assigned at random, weighted by the probability of giving birth in a given month. We calculated the percentage of time during which the delivery room is empty for the busiest month (January in the Sierra Leone data) and the least busy month (November in the Sierra Leone data).

### Zanzibar analysis

We used data on births and Caesarean sections per facility, number of beds and staffing levels at am and pm times from 37 EmOC facilities in Zanzibar for a representative month in 2014 (data have been collected by the Pemba Public Health Laboratory and can be made available on request). Staffing levels included information on SBAs only, not on medical doctors or nurses. Data on facilities were collected during an assessment across maternity units in Zanzibar, commissioned by the MoH in 2013 to inform a quality improvement process for maternity wards (further details on methodology and findings are reported elsewhere^13^). Seven of the facilities carried out Caesarean sections in 2014. We assumed that these facilities were CEmOCs, while all other facilities were BEmOCs. Two of the 37 facilities did not report any births in 2014 and were excluded from the analysis. Births requiring a Caesarean section were classified as complicated, while all other births were assumed to be uncomplicated. In total, 2943 births were recorded in EmOC facilities in Zanzibar over this average month. In 2013, the year of the facility survey, 48 763 births were reported in Zanzibar.^16^

Similar to the WHO benchmark analysis, we assigned each woman a time for presentation in a maternity facility and a complicated or uncomplicated birth at random. The proportion of births ending in Caesarean sections reported in the Zanzibar data set was 11.9% which is close to the 15% complicated births assumed in the World Health Report.^2^ To account for complicated births other than Caesarean sections, we assume a probability of 15% for a birth to be complicated. We assume that the number of births reported for each facility in the data set over a month are representative. Consequently, the probability of a woman presenting at each facility depends on the monthly births reported for this facility. However, we assumed that women with complicated births only give birth at one of the facilities identified as CEmOCs, because complicated births were not reported in other facilities.

The LOS in the delivery room for each woman is drawn from the same gamma distribution as for the WHO benchmark scenario. The LOS in the maternity ward for each woman is drawn from a gamma distribution fitted to data from the DHS for Zanzibar.^10^

### Zanzibar analysis with seasonality

We extracted data on the number of births by month in Zanzibar from the 2015–2016 District Health Survey for Tanzania. We obtained probability weights for giving birth in a specified month by dividing the number of births reported in each month by the total annual number of births. In the simulation, we randomly assigned a date and time of presentation of each pregnant woman at a facility weighted by the probability of giving birth in each month. We calculated the percentage of time during which the delivery room is empty for the busiest month (June in Zanzibar) and the least busy month (January in Zanzibar). We also calculated the percentage of time during which the capacity of each facility is exceeded in terms of beds and staffing. We assumed that each SBA should look after only one woman in labour at once, as recommended in the UK,^17^ Australia^18^ and New Zealand,^19^ and under discussion in Germany.^20 21^

### Patient and public involvement

Patients and the general public were not involved in data collection or analysis of this study.

## Results

### WHO benchmark analysis

**Figure 1:**
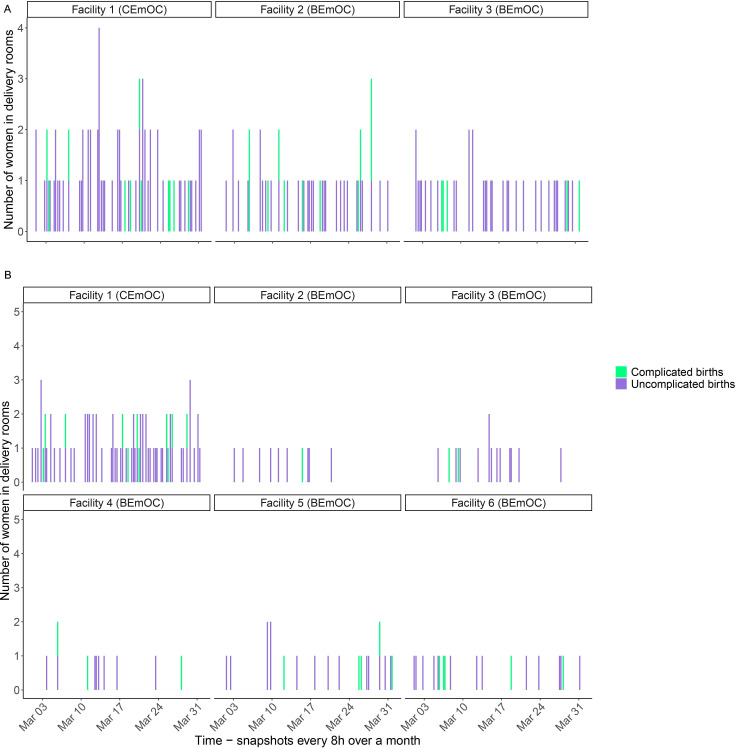
WHO benchmark analysis. Number of women in delivery rooms (panels a and b) in different facilities at the beginning of each 8 hour shift over a representative month, showing complicated and uncomplicated births. (A) shows results for scenario A; (B) shows results for scenario B. WHO benchmark scenario A: one CEmOC staffed by 10 SBAs and two BEmOCs staffed by five skilled birth attendants (SBAs) each; WHO benchmark scenario B: one CEmOC staffed by 10 SBAs and five BEmOCs staffed by two SBAs each. Facility 1 in each scenario is a CEmOC. All other facilities are BEmOCs. Snapshots are spaced evenly. This means that if there are extended gaps between bars, these are shifts without any births in the facility. BEmOC, Basic Emergency Obstetric Care Centre; CEmOC, Comprehensive Emergency Obstetric Care Centre.

Figure 1A,B show the number of women in the delivery room in each facility in a single model run of the stochastic simulation for both scenarios of the WHO benchmark analysis. This model run has been selected at random and highlights the stochastic variation in chance events. SBAs working in the small BEmOC facilities in scenario B only see a few complicated births over a month. If women can feasibly travel to a CEmOC rather than a BEmOC (as may be the case in more densely populated areas where facilities are close to one another), then midwives in the BEmOC facilities may well see very few complicated cases and start to lose their skills to recognise signs for high-risk conditions. Online supplemental appendix 2 figures S3A,B show the number of women in postpartum beds over a month, for the same scenarios. Note that the proportion of women who have experienced complicated births is larger in postpartum beds because their expected LOS is longer.

**Figure 2:**
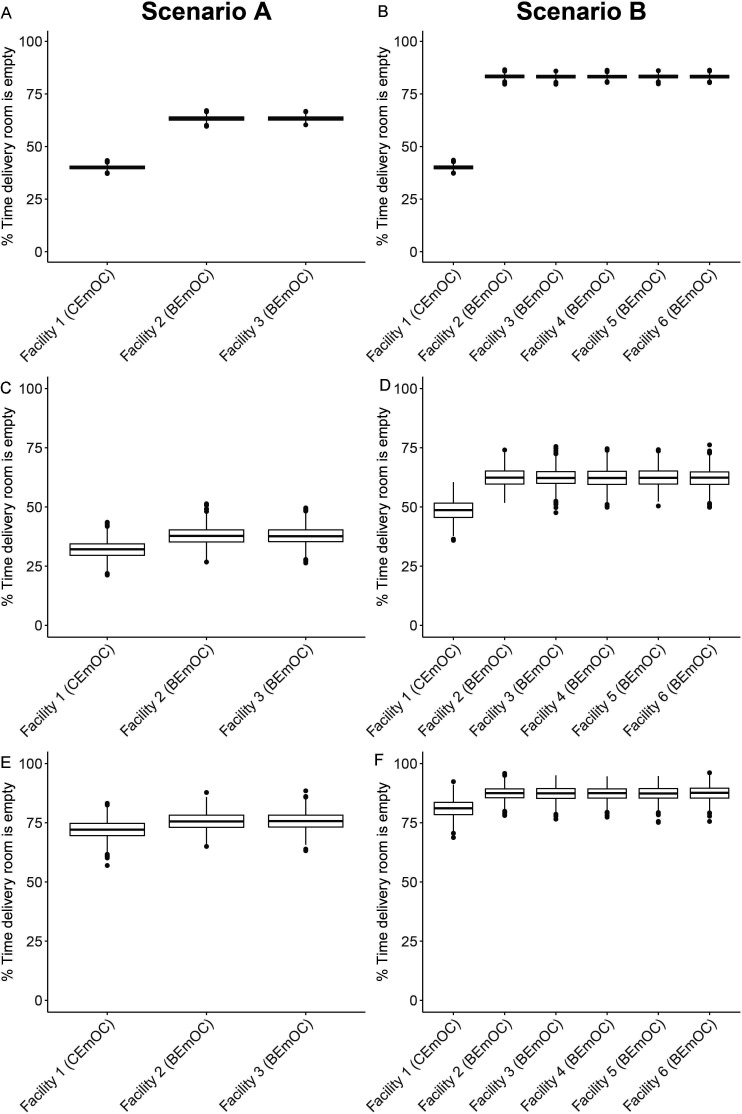
WHO benchmark analysis. Percentage of time over a year in which delivery rooms are empty. Results from 1000 iterations of the stochastic simulation. Boxes: IQR. Whiskers extend to 1.5 times the IQR. Dots are outliers. WHO benchmark scenario A: one CEmOC staffed by 10 SBAs and two BEmOCs staffed by five skilled birth attendants (SBAs) each; WHO benchmark scenario B: one CEmOC staffed by 10 SBAs and five BEmOCs staffed by two SBAs each. Facility 1 in each scenario is a CEmOC. All other facilities are BEmOCs. BEmOC, Basic Emergency Obstetric Care Centre; CEmOC, Comprehensive Emergency Obstetric Care Centre. (A,B) base case, assuming no birth seasonality. (C–F) results for a hypothetical setting with marked birth seasonality (see Methods for details). Results are percentage of time a facility is empty over the busiest month (January, C and D) and the least busy month (November, E and F).

Figure 2A,B show the summary results from running the stochastic simulation 1000 times to understand the general behaviour of the model. The simulation samples reflected our model inputs well. Across the 1000 simulations generated, the mean and median proportion of deliveries that were complicated were both 15.0% and for duration in the delivery room, the mean of the means for 3600 births across 1000 iterations was 4.48 hours and the median of the medians of 3600 births across 1000 iterations was 3.92 hours, in close agreement with our inputs for these parameters, shown in online supplemental appendix 1 table S1.

Figure 2A,B show the percentage of time over a year in which delivery rooms in different facilities are empty for the two WHO benchmark scenarios. For both scenarios, CEmOCs are busier than BEmOCs; they are empty a mean 40.1% of the time (median 40.1%, 95% credible interval (95% CrI) 38.5%–41.6% for scenario A, 38.4%–41.7% for scenario B), while BEmOCs are empty a mean 63.3% of the time (median 63.3%, 95%CrI 61.5%–65.1%) for scenario A (two BEmOCs), and 83.3% of the time (median 83.8%, 95%CrI 81.8%–84.9%) for scenario B (four BEmOCs). Maintaining a larger number of smaller BEmOCs (scenario B) therefore increases the percentage of time in which delivery rooms in BEmOCs are empty relative to a scenario with fewer and larger BEmOCs. Although smaller BEmOCs are empty more frequently in the simulated scenarios, individual SBAs in these BEmOCs see the same number of deliveries per year as SBAs in larger CEmOCs because the staffing ratio in smaller facilities is lower (online supplemental appendix 2 figures S4A,B). This is because we assume that the probability for a pregnant woman to give birth at a CEmOC or in any of the BEmOC facilities is 50%. Under the simulated conditions, SBAs in both CEmOCs and BEmOCs see approximately 175 deliveries per year, the number recommended by WHO.^2^

On average, 1176 pregnant women enter labour at night (SD 28.65) for a ‘typical’ district over 1 year. This is roughly a third of total births which is intuitive if time of day of birth is random and there are three daily shifts. Out of these 1176 births on average 112 (SD 10.58) can be expected to be complicated. In scenario B, BEmOCs would not be staffed at night. Therefore, women entering labour at night would have to attend the CEmOC facility which would increase delivery room occupancy at night and in the early morning.

### WHO benchmark analysis with seasonality

Over a year, the average results for the WHO benchmark analysis with seasonality do not differ from the analysis in which seasonality is ignored (online supplemental appendix 2 figures S4C,D and S5). However, there are noticeable differences between the busiest (January) and least busy month (November) (figure 2C–F). In November, delivery rooms are empty most of the time. Small BEmOCs in scenario B are empty 95% of the time (figure 2F). In contrast, in January delivery rooms in scenario A are busy most of the time (figure 2C). In scenario B, with more smaller BEmOC facilities, delivery rooms are still empty at least 50% of the time (figure 2D,F).

### Zanzibar analysis

**Figure 3:**
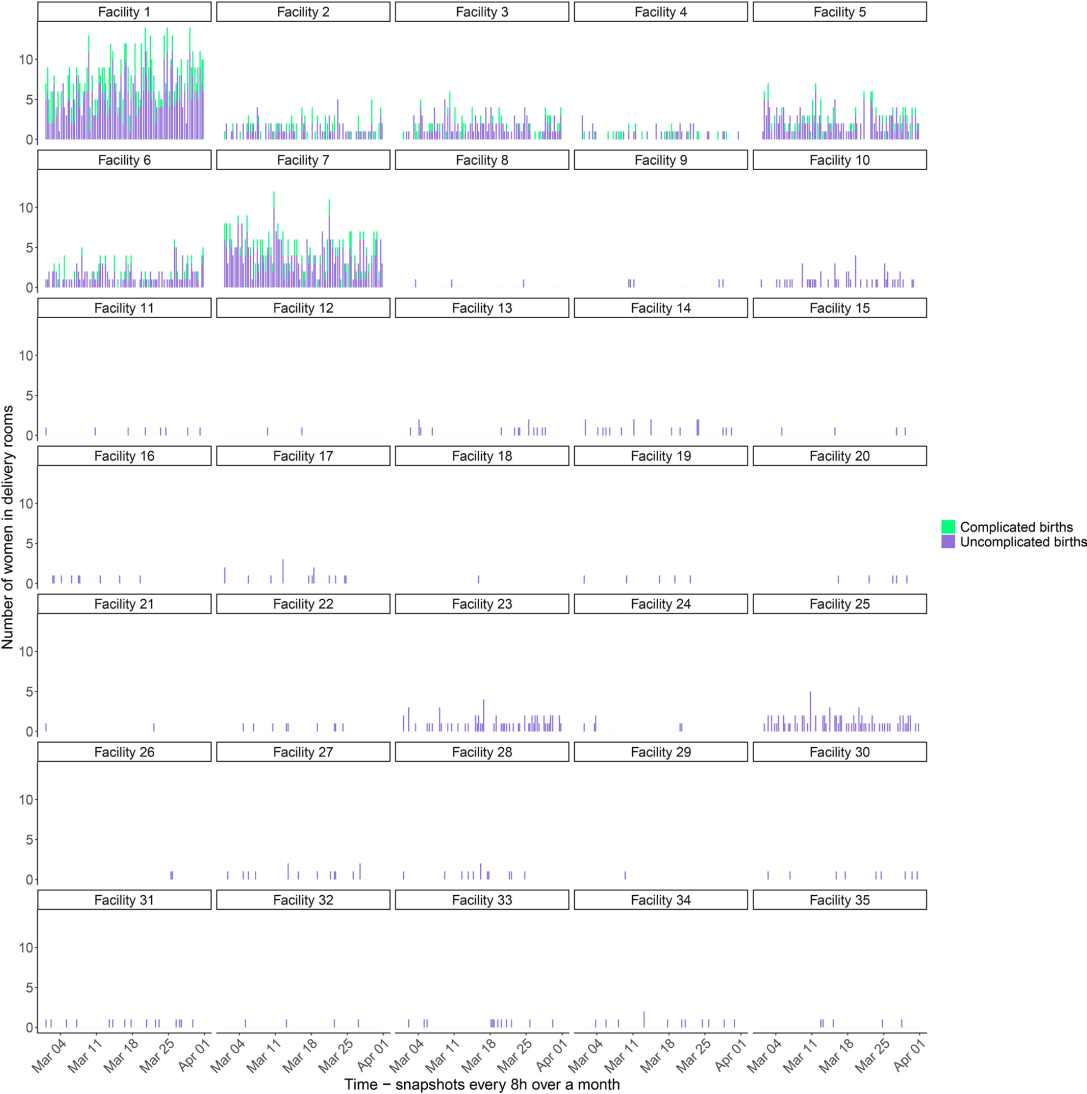
Zanzibar analysis. Simulated number of women in delivery rooms in 35 EmOC facilities in Zanzibar in 8 hour snapshots over a representative month, showing complicated and uncomplicated births. Seven facilities (facilities 1 to 7) undertake Caesarean sections. Complicated births only occur in facilities that have been identified as CEmOCs and that are able to carry out Caesarean sections. Snapshots are spaced evenly. This means that if there are extended gaps between bars, these are shifts without any births in the facility. BEmOC, Basic Emergency Obstetric Care Centre; CEmOC, Comprehensive Emergency Obstetric Care Centre.

Figure 3 shows the number of women in delivery rooms in 35 EmOC facilities in Zanzibar in 8 hour snapshots over 1 month, estimated using our model, calibrated using empirical data on number of deliveries over a representative month in all facilities on the island. The same stochasticity is observed in this real-world scenario as in the generic WHO benchmark analysis. However, the differences in the numbers of women attending different facilities are more striking. The majority of facilities are small and rarely see more than one woman in the delivery room at a time, whereas in the largest facility (facility 1) at times, there are more than 10 women in the delivery room at once. A similar pattern can be observed for postpartum bed occupancy (online supplemental appendix 2 figure S6). However, in CEmOCs women who have experienced complicated births are over-represented, occupying more than half of postpartum beds, because their average LOS is longer.

**Figure 4:**
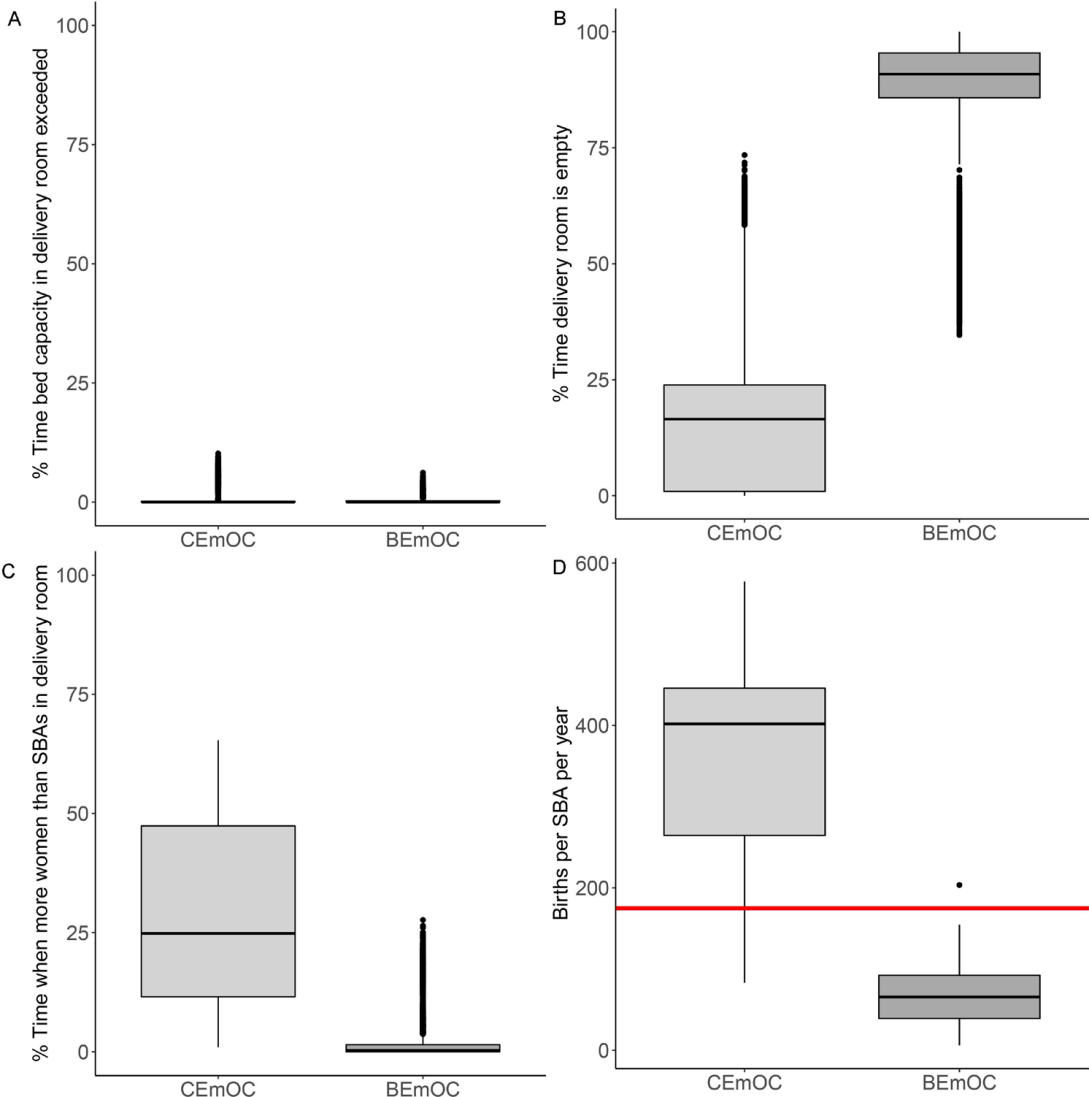
Zanzibar analysis. Occupancy statistics for 7 CEmOC and 28 BEmOC facilities in Zanzibar. Results from 1000 iterations of the stochastic simulation. Boxes: IQR. Whiskers extend to 1.5 times the IQR. Dots are outliers. (A) Percentage of time over a representative month when bed capacity in delivery rooms is exceeded. (B) Percentage of time when delivery rooms are empty. (C) Percentage of time when more women than SBAs are in a delivery room. (D) Births per SBA per year. The red line indicates the 175 births per year recommended by WHO.^2^ BEmOC, Basic Emergency Obstetric Care Centre; CEmOC, Comprehensive Emergency Obstetric Care Centre; SBA, skilled birth attendant.

Delivery room capacity in terms of beds is rarely exceeded and if so, then only in smaller CEmOCs and BEmOCs (figure 4A, online supplemental appendix 2 figure S7A). However, these rare events are important because they can lead to a deterioration of quality of care for affected women. Figure 4B shows that BEmOCs are empty most of the time. Smaller BEmOCs are empty more than 80% of the time (online supplemental appendix 2 figure S7B). The larger a CEmOC facility is (ie, the larger the mean number of women attending a facility within a month), the less likely it is that the delivery room is unoccupied. The largest CEmOC facility is almost never empty (facility 1, online supplemental appendix 2 figure S7B). The ratio of women in labour to SBAs is exceeded on average 25% of the time in CEmOCs (figure 4C), ranging from 5% to 56% of the time in individual facilities (online supplemental appendix 2 figure S7C). Staff capacity in BEmOCs is exceeded less than 5% of the time on average, but can be exceeded up to 20% of the time in some BEmOCs (online supplemental appendix 2 figure S7C).

As figure 4D shows, SBAs working in busy CEmOCs on average attend more than the 175 births per year in Zanzibar, in some facilities three times that number (online supplemental appendix 2 figure S8). This means that the overall workload on SBAs in these facilities may be excessive. Conversely, SBAs in small BEmOCs on average attend considerably fewer than 175 births per year. This means that in the least frequented facilities SBAs may not see enough births per year to maintain adequate training.

### Zanzibar analysis with seasonality

**Figure 5:**
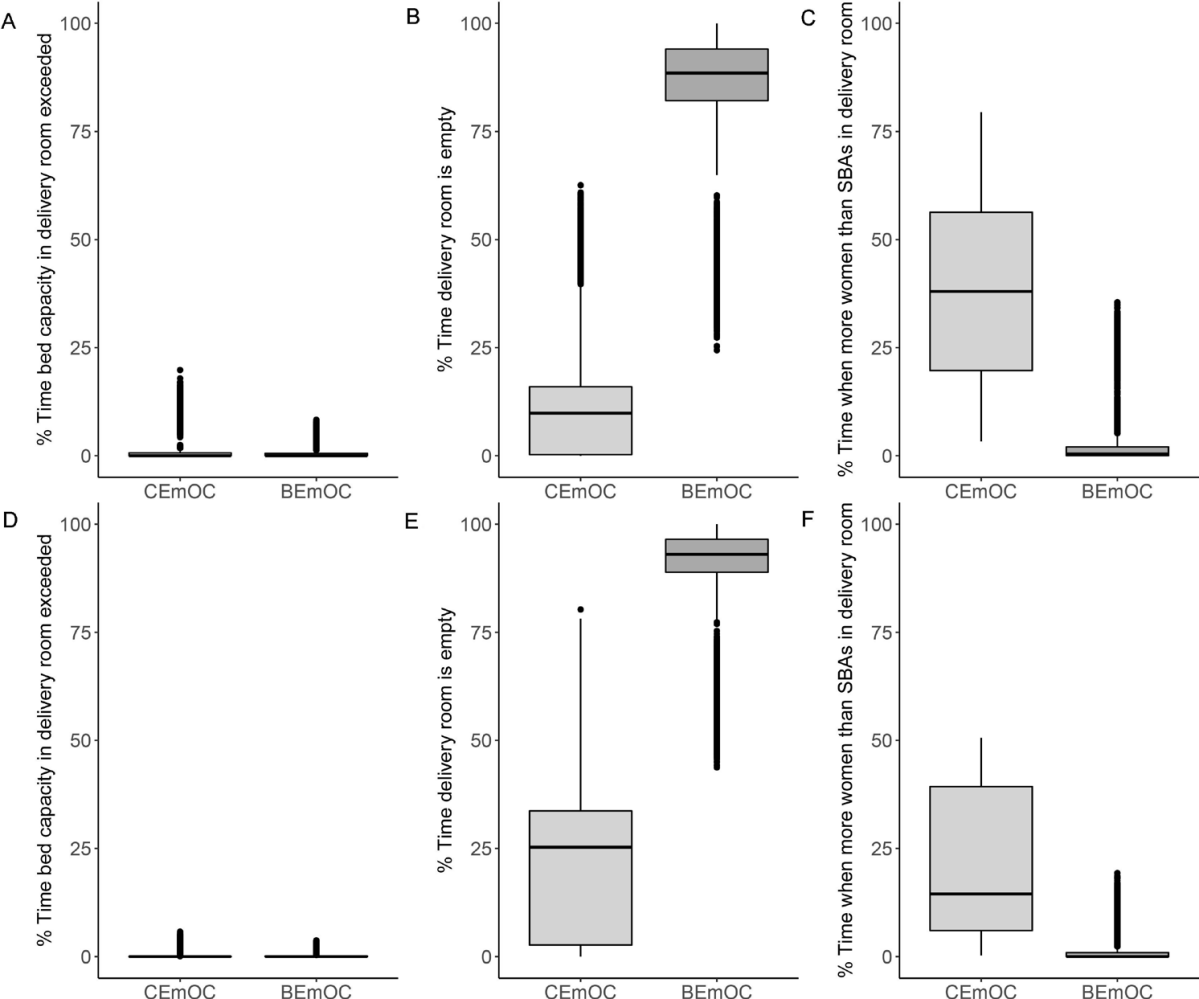
Zanzibar analysis with seasonality. Occupancy statistics for 7 CEmOC and 28 BEmOC facilities in Zanzibar in the least busy month (January, A–C) and the busiest month (June, D–F). Results from 1000 iterations of the stochastic simulation. Boxes: IQR. Whiskers extend to 1.5 times the IQR. Dots are outliers. A and D: percentage of time when bed capacity in delivery rooms is exceeded; B and E: percentage of time when delivery rooms are empty; C and F: percentage of time when more women than SBAs are in a delivery room. BEmOC, Basic Emergency Obstetric Care Centre; CEmOC, Comprehensive Emergency Obstetric Care Centre; SBA, skilled birth attendant.

Similar to the WHO benchmark analysis, the average results over a year do not change significantly if birth seasonality is considered in the Zanzibar analysis (online supplemental appendix 2 figure S9). However, there are important differences in delivery room occupancy between the busiest month (June) and the least busy month (January) (figure 5, online supplemental appendix 2 figures S10,11). While delivery room capacity in terms of beds is rarely exceeded in either January or June, the risk of staff capacity being exceeded in June is double the risk in January. In particular, CEmOC facilities 1 and 7 have on average a greater than 50% risk of exceeding staff capacity in June (online supplemental appendix 2 figure S11C), and CEmOC facility 2 has an average risk of 10% of exceeding bed capacity in June (online supplemental appendix 2 figure S11A). A few BEmoCs (facilities 21, 24, 27) have a greater than 5% risk of exceeding staff capacity in both January and June despite being empty most of the time (online supplemental appendix 2 figures S10B,C and S11B,C).

## Discussion

Our analysis illustrates various conflicting pressures with which health systems and particularly maternal healthcare providers must contend. The WHO benchmark analysis demonstrated that while there are extended periods of redundancy (empty delivery rooms), facilities are very rarely over capacity, which is the key outcome in terms of minimising risk to women and their babies. SBAs at facilities with empty delivery rooms are likely to be deployed usefully elsewhere (such as in the maternity ward). Our analysis also shows that meeting SBA coverage per population benchmarks is not sufficient to ensure high quality of care for women in labour at all times. A review from 2016 found that 64 out of 132 countries do not meet the minimum target of providing 23 SBAs, nurses and doctors per 10 000 population.^11^ Guidelines in some countries, for example, the UK, recommend hiring an additional 17.5%–25% to allow for absence due to sickness, study or annual leave.^22^ Our analysis illustrates that because pregnant women do not enter delivery rooms at a regular, predictable rate, facilities need to plan for peaks when more women are in the delivery room than on average. In the Zanzibar analysis, we find that delivery rooms are rarely overcrowded in terms of bed occupancy, but that at peak times, too few SBAs may be available in the delivery room to provide one-to-one care for women in labour at large and busy facilities (figure 5A,C). Consequently, SBA coverage targets may not be sufficient to ensure high quality of care standards are maintained during peak hours.

There are no universal guidelines on the ratio of SBAs to women in labour in the delivery room.^12^ Many high-income countries recommend one-to-one care during labour.^17^,^19^ California issued guidelines that one SBA should not look after more than two women in labour at the same time.^23^ Nevertheless, even in high-income settings, these targets are not always reached. For example, a representative survey of 1692 midwives in Germany in 2015 found that 46% of midwives regularly look after three women at once in the delivery room, and 5% of midwives look after more than four women at once.^24^ In the Zanzibar analysis, one-to-one care for women in labour could be met only 40% of the time in the busiest CEmOC facilities and in only 25% of the time in the busiest month when birth seasonality was considered (online supplemental appendix 2 figures S7, S10, S11). Some small BEmOC facilities also exceeded a one-to-one SBA: women in labour ratio which indicates that they may be understaffed to adequately serve the population in their catchment areas. To alleviate pressure on specialist staff on obstetric wards, some countries, for example, the UK, recommend increasing the number of home births and births in midwifery centres.^25^ However, this is only possible for births that are expected to proceed without complications or with an efficient referral system where midwifery centres are near hospitals. A universal standard for the SBA-to-woman ratio would inform our analysis and facilitate health policy planning by allowing us to predict the frequency of falling below a minimum threshold of a staffing policy, and linking that to the impact on patient care.

Our model has some methodological limitations, including some simplifying assumptions that affect our results. We assume that there are no referrals from BEmOCs to CEmOCs. In reality, women with unexpected complications who present at a BEmOC facility but need comprehensive care may be referred to a CEmOC facility, although referral systems tend to be weak in resource-poor settings. In low-income countries, only 21% of complicated births happen in EmOCs and this only increases to 32% in middle-income settings.^14^ Referrals may exacerbate crowding and pressure on staff in CEmOCs. Therefore, if a referral system is implemented, staffing requirements in CEmOCs should be reviewed. Complications that may arise during delivery are not always predictable. Smaller BEmOCs where SBAs see very few complicated births may lack the experience to recognise complications early and initiate a referral to a CEmOC facility if required. For example, the 2016 review of 132 countries found that in lower- and middle-income countries, more than 70% of birth facilities tend to be low-volume, seeing less than 500 births per year.^14^ Note that in the Zanzibar analysis, we assumed that all complicated births would present at CEmOCs (figure 3), because data on complicated births in BEmOCs were not collected as part of the 2013 survey.

As with many maternal health outcomes, there is some uncertainty in our model parameters because of difficulties in measurement, meaning that few estimates exist in the published literature (for example, the proportion of deliveries with complications). We have necessarily made assumptions for some parameters, such as the duration in the delivery room for women with complicated deliveries, which will likely vary substantially based on the nature of the complication. However, where data exist to inform our model estimates, we have robustly fitted our parameter values to generate scenarios as close to real-world as possible. The number of SBAs required to guarantee quality care may vary substantially depending on the complication, but as mentioned above, there are no universal guidelines on the ratio of SBAs to women in labour in the delivery room, which may well be because it is so difficult to estimate, given this heterogeneity. We assume that discharge times are independent of circumstances at the facility, such as crowding. In reality, women may be discharged early if facilities are crowded. This would reduce peak occupancy levels in maternity wards, but early discharge may pose a risk for maternal and child health.^14^ Finally, while we evaluate what numbers of CEmOC and BemOC facilities may be optimal, this does not take into account accessibility to these facilities for the modelled population, where access for those living in remote, rural areas may be an issue. Spatial modelling, taking into account population density and perhaps even variation in fertility between urban and rural regions, and the use of maternity waiting homes close to facilities, would be the next logical step for this analysis. Nonetheless, this study effectively demonstrates that event stochasticity is important for health policy planning and risks periods of both redundancy and over-capacity that need to be considered more fully.

Delivery rooms in small facilities are empty most of the time in both the WHO benchmark analysis and the Zanzibar analysis. In the WHO benchmark scenarios, SBAs in both CEmOCs and BEmOCs see about the recommended 175 births per year. With the exception of a few facilities in the Zanzibar analysis, pressure on staff in small BEmOCs tends to be low. This indicates that under some circumstances, a smaller number of better staffed facilities may be preferable to a greater number of small facilities that are empty most of the time, both in terms of efficiency and in providing sufficient training hours to staff. Maintaining a large number of small BEmOCs may be inefficient but necessary to meet recommended standards of access to facilities. WHO recommends that all pregnant women should be able to reach an EmOC facility within 2–3 hours of their home.^26^ However, recent studies from Nigeria indicate that the risk of maternal mortality in resource-poor settings increases beyond 10 km from a CEmOC facility, even if travel times are within 30 min.^27 28^ Travel time does not only depend on absolute distance but also on other factors, such as access to transport, road quality and congestion. Thus, closing small facilities may not be an option if travel time for pregnant women increases. Moreover, small BEmOCs do not only offer obstetric care but also vital antenatal care.^29^,^31^

Our simulations show that small facilities tend to be empty for a large proportion of time, both in the WHO benchmark scenarios and in the real-world case study of Zanzibar (figures2  4). As a consequence, SBAs at small facilities may not see enough births or complicated births per year to develop or maintain skills and experience. This could potentially be mitigated by sharing of staff between facilities. According to the WHO benchmark analysis, about a third of women are expected to arrive in EmOC facilities at night. In a typical SSA district, more than a 100 of these women are expected to experience complications during birth that require comprehensive emergency obstetric care. Very small facilities with insufficient number of SBAs to staff delivery rooms at all hours may not be able to provide adequate care for these women. For example, a 2012–2013 survey in Zanzibar found that only 81% of facilities could staff both am and pm shifts with SBAs, while 100% are required for EmOC facilities.^32^ In remote areas, where increasing the number of staff may be difficult, flexible shift times with SBAs on call may help to ensure that women in labour have access to at least basic obstetric care.

### Conclusion

Our simulation analysis shows that considering maternal health benchmarks based on average estimates of women presenting at obstetric care facilities may be insufficient to provide adequate standard of care during peak times, when crowding in the delivery room occurs. Crowding or lumping is an intrinsic feature of systems with random events (births) and waiting times (time in the delivery room). Stochastic individual-based models capture important features of these systems and, combined with additional contextual information, can be used to optimise obstetric care provisions, for example, to support decisions on maintaining/closing or upgrading individual facilities, required resourcing and staffing.

## supplementary material

10.1136/bmjph-2023-000212 online supplemental file 1

10.1136/bmjph-2023-000212 online supplemental file 2

## Data Availability

Data are available in a public, open access repository.
