# Supplementary material for: Do current maternal health staffing and bed occupancy benchmarks work in practice? Results from a simulation model
Source: BMJ Public Health. 2024 Jan 19;2(1):e000212. doi: 10.1136/bmjph-2023-000212 (PMC11816098; doi:10.1136/bmjph-2023-000212)
Supplement: online supplemental file 1 [file bmjph-2-1-s001.pdf]

Appendix 1: Supplementary Methods

Baggaley et al

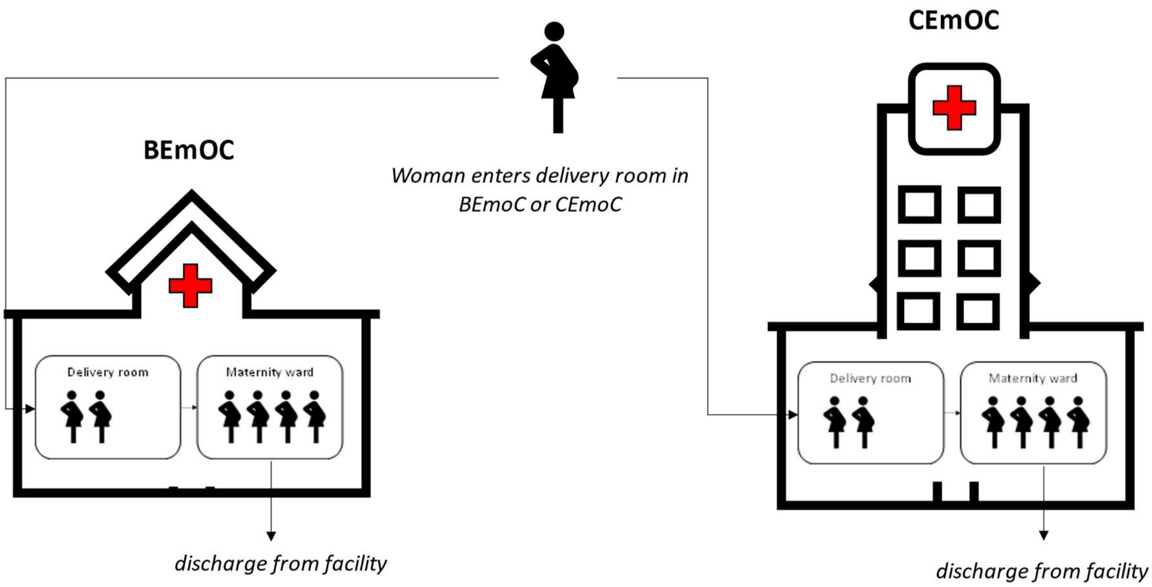

Figure S1: Schematic representation of women entering and being discharged from emergency obstetric care facilities. Women enter delivery rooms in BEmOC (basic emergency obstetric care) or CEmOC (comprehensive emergency obstetric care) facilities. As complications and need for CEmOC are hard to predict, women choose facilities based on prior preferences. If a woman presenting at a BEmOC facility requires comprehensive care, ideally she would be referred to a CEmOC facility. However, referral systems are weak in resource-poor setting. For simplicity, we do not consider referrals in the simulations.

Table S1: Description and values of model inputs.

**Parameter**	**Value**	**Source**
Deliveries per year	3600 deliveries	World Health Report 2005[1]
Proportion of deliveries with complications	15%	Bang et al 2004;[2] World Health Report 2005[1]
Minimum duration in delivery room	1 hour	Assumption
Duration in delivery room for uncomplicated labour, data from Nigeria	Mean 4.19 hours<br>Median 3.75 hours	Ijaiya et al[3]
Gamma distribution parameters for LOS in delivery room: uncomplicated deliveries, data from Nigeria, hours	Shape 3.37<br>Scale 1.23	

Factor increase in labour duration with complications	1.5	Assumption
Likelihood of delivery in a CEmOC facility – complicated deliveries	50%	Assumption based on Campbell et al 2016[4]
Likelihood of delivery in a CEmOC facility – uncomplicated deliveries	50%	Assumption based on Campbell et al 2016[4]
Gamma distribution parameters for LOS in maternity ward: uncomplicated deliveries, Zanzibar, hours	Shape (alpha) 1.11<br>Scale (beta) 12.08	Tanzania DHS 2015-16 (Zanzibar facility data only)[5]
Gamma distribution parameters for LOS in maternity ward: complicated deliveries, Zanzibar, hours	Shape (alpha) 2.02<br>Scale (beta) 57.80	Tanzania DHS 2015-16 (Zanzibar facility data only)[5]
Gamma distribution parameters for LOS in maternity ward: uncomplicated deliveries, sub-Saharan Africa (WHO benchmark analysis), hours	Shape (alpha) 1.1<br>Scale (beta) 50.2	Campbell et al[6]
Gamma distribution parameters for LOS in maternity ward: complicated deliveries, sub-Saharan Africa (WHO benchmark analysis), hours	Shape (alpha) 1.2<br>Scale (beta) 136.0	Campbell et al[6]
Minimum recommended length of stay postpartum for a uncomplicated labour	≥24 hours	Based on a WHO recommendation for uncomplicated vaginal deliveries[7]
Minimum recommended length of stay postpartum for a complicated labour	≥72 hours	Campbell et al[6]

CEmOC – Comprehensive Emergency Obstetric Care Centre; DHS – Demographic and Health Surveys; LOS – length of stay.

Estimating length of stay in the postpartum ward

We used data from Campbell et al[6] which in turn used Demographic and Health Surveys (DHS) data. For the evaluation of the WHO benchmarks, we limited our analysis to all included sub Saharan African countries (n=14 Benin, Gabon, Ghana, Kenya, Lesotho, Liberia, Madagascar, Namibia, Nigeria, Sao Tome and Principe, Sierra Leone, Swaziland, Uganda and Zambia). For the evaluation of Zanzibar data, we used Tanzania DHS data looking at data from Zanzibar only.[5] Length of stay has been shown to be highly skewed (Campbell et al[6] Figure 1). We used Cullen and Frey graphs from the R fitdistrplus package to confirm that the gamma distribution was appropriate to model the data. Therefore we fitted gamma distributions to the data using maximum likelihood. We conducted the analysis using R version 3.0.3 (2014-03-06).

Our model separates delivery type into complicated and uncomplicated; we used the delivery modes Caesarean section and vaginal birth from Campbell et al[6] as proxies for these. We excluded births where length of stay was recorded as greater than three weeks, as performed by Campbell et al.[6]

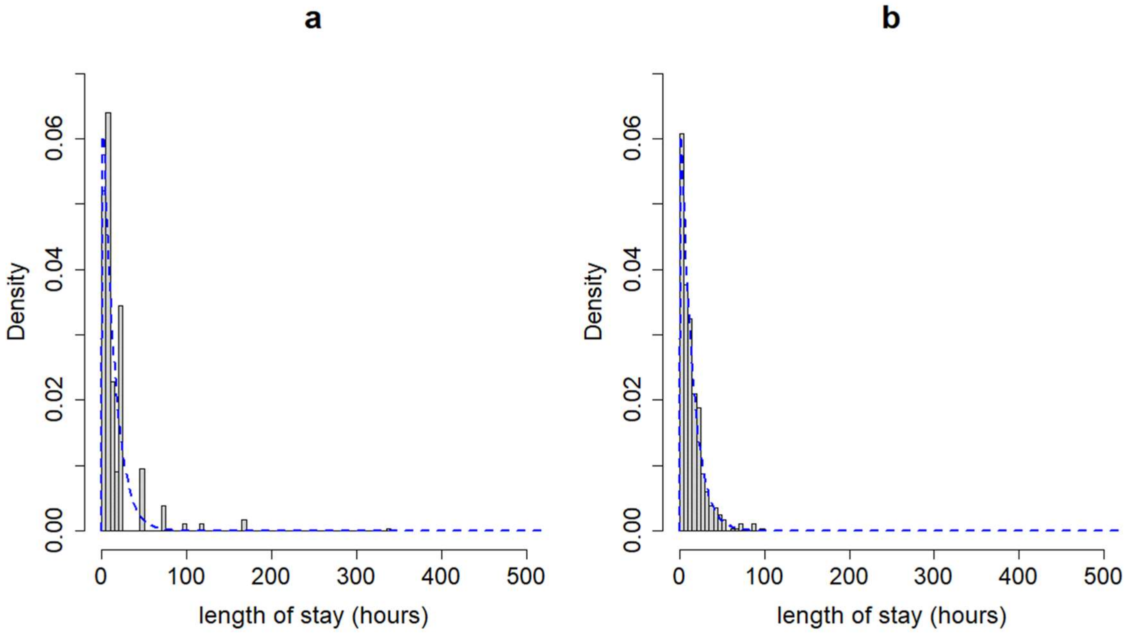

Figure S2 Comparison of DHS data (panel a) and random sample of lengths of stay drawn using the gamma fitted probability density function (panel b). Data shown are vaginal births, Zanzibar, Tanzania.[5] Dotted lines are the gamma probability density function fit to the DHS data using maximum likelihood. Outliers with length of stay in EmOCs of more than 20 days have been removed.

Estimating length of stay in the delivery room

We used data from Ijaiya et al[3] on time from admission to delivery at University of Ilorin Teaching Hospital, Ilorin, Nigeria, for vaginal deliveries, which we used as a proxy for uncomplicated deliveries. Mean (SD) duration between and admission and delivery was 4.19 (3.03) hours, median 3.75 hours. We fitted a gamma distribution using least squares fitting to the median duration using the Solver

function in MS Excel. Best fit shape (alpha) and scale (beta) parameters were 3.37 and 1.23 respectively, which when used to generate 10,000 values according to this distribution, produced a median of 3.78, mean 4.17 and SD 2.27, which we were satisfied was a reasonable fit. We could not find information on admission to delivery for deliveries ending in Caesarean, so arbitrarily selected a 1.5-fold increase in duration for complicated deliveries compared to uncomplicated deliveries. We used the same gamma distribution as for complicated deliveries to generate durations in the delivery room, and multiplied each of these by 1.5 to generate our distribution of estimates.

References

1. WHO. The World Health Report 2005. Make every mother and child count. Published 16th June 2005. Available from: https://www.who.int/publications/i/item/9241562900 Accessed 28th August 2022. 2005.

2. Bang RA, Bang AT, Reddy MH, Deshmukh MD, Baitule SB, Filippi V. Maternal morbidity during labour and the puerperium in rural homes and the need for medical attention: A prospective observational study in Gadchiroli, India. *BJOG* 2004; **111**(3): 231-8.

3. Ijaiya MA, Adesina KT, Raji HO, et al. Duration of labor with spontaneous onset at the University of Ilorin Teaching Hospital, Ilorin, Nigeria. *Ann Afr Med* 2011; **10**(2): 115-9.

4. Campbell OM, Calvert C, Testa A, et al. The scale, scope, coverage, and capability of childbirth care. *Lancet* 2016; **388**(10056): 2193-208.

5. DHS. Demographic and Health Surveys Program. Tanzania Demographic and Health Survey and Malaria Indicator Survey 2015-2016 [Dataset]. TZIR7BFL.DTA. Ministry of Health, Community Development, Gender, Elderly and Children (MoHCDGEC) [Tanzania Mainland], Ministry of Health (MoH) [Zanzibar], National Bureau of Statistics (NBS), Office of the Chief Government Statistician (OCGS), and ICF. 2016. Tanzania Demographic and Health Survey and Malaria Indicator Survey (TDHS-MIS) 2015-16. Dar es Salaam, Tanzania, [Producers] and Rockville, Maryland, USA: MoHCDGEC, MoH, NBS, OCGS, and ICF [Distributor]. Available from: dhsprogram.com Accessed 31 October 2022. 2016.

6. Campbell OM, Cegolon L, Macleod D, Benova L. Length of Stay After Childbirth in 92 Countries and Associated Factors in 30 Low- and Middle-Income Countries: Compilation of Reported Data and a Cross-sectional Analysis from Nationally Representative Surveys. *PLoS Med* 2016; **13**(3): e1001972.

7. WHO. World Health Organization. WHO recommendations on postnatal care of the mother and newborn. Geneva: World Health Organisation. Available at: https://www.ncbi.nlm.nih.gov/books/NBK190090/#:~:text=2013%20WHO%20Recommendations%20on%20postnatal%20care&text=After%20an%20uncomplicated%20vaginal%20birth,least%2024%20hours%20after%20birth. Accessed 28th August 2022. 2013.
