# Supplementary material for: Do current maternal health staffing and bed occupancy benchmarks work in practice? Results from a simulation model
Source: BMJ Public Health. 2024 Jan 19;2(1):e000212. doi: 10.1136/bmjph-2023-000212 (PMC11816098; doi:10.1136/bmjph-2023-000212)
Supplement: online supplemental file 2 [file bmjph-2-1-s002.pdf]

Appendix 2: Supplementary Results

Baggaley et al

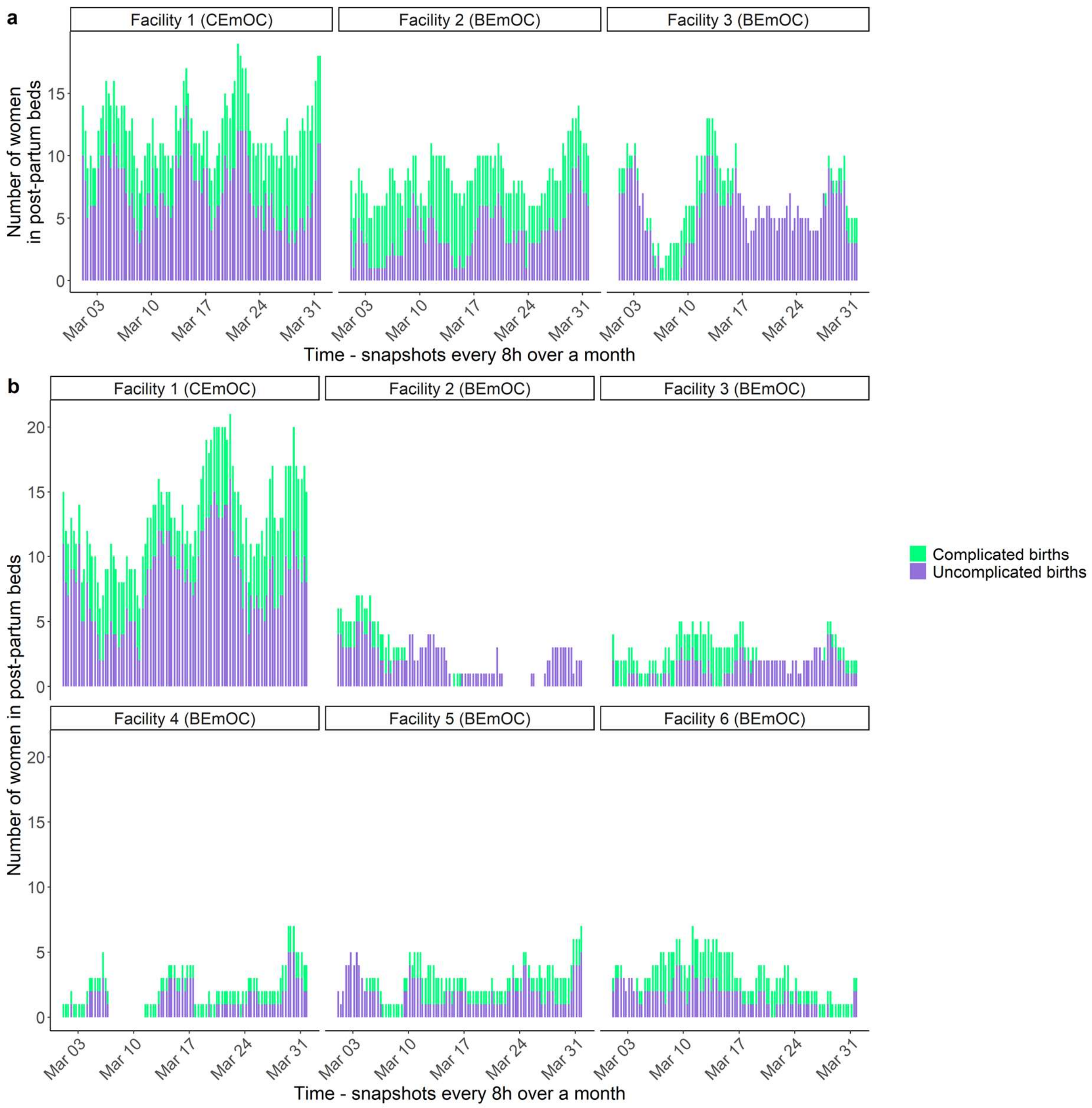

Figure S3: WHO Benchmark analysis. Number of women in post-partum beds in different facilities at the beginning of each eight-hour shift over a representative month, in Scenario A and B, showing complicated and uncomplicated births. Facility 1 in each scenario is a CEmOC. All other facilities are BEmOCs. Snapshots are spaced evenly. See Methods in main text for description of different scenarios. BEmOC – Basic Emergency Obstetric Care Centre; CEmOC – Comprehensive Emergency Obstetric Care Centre.

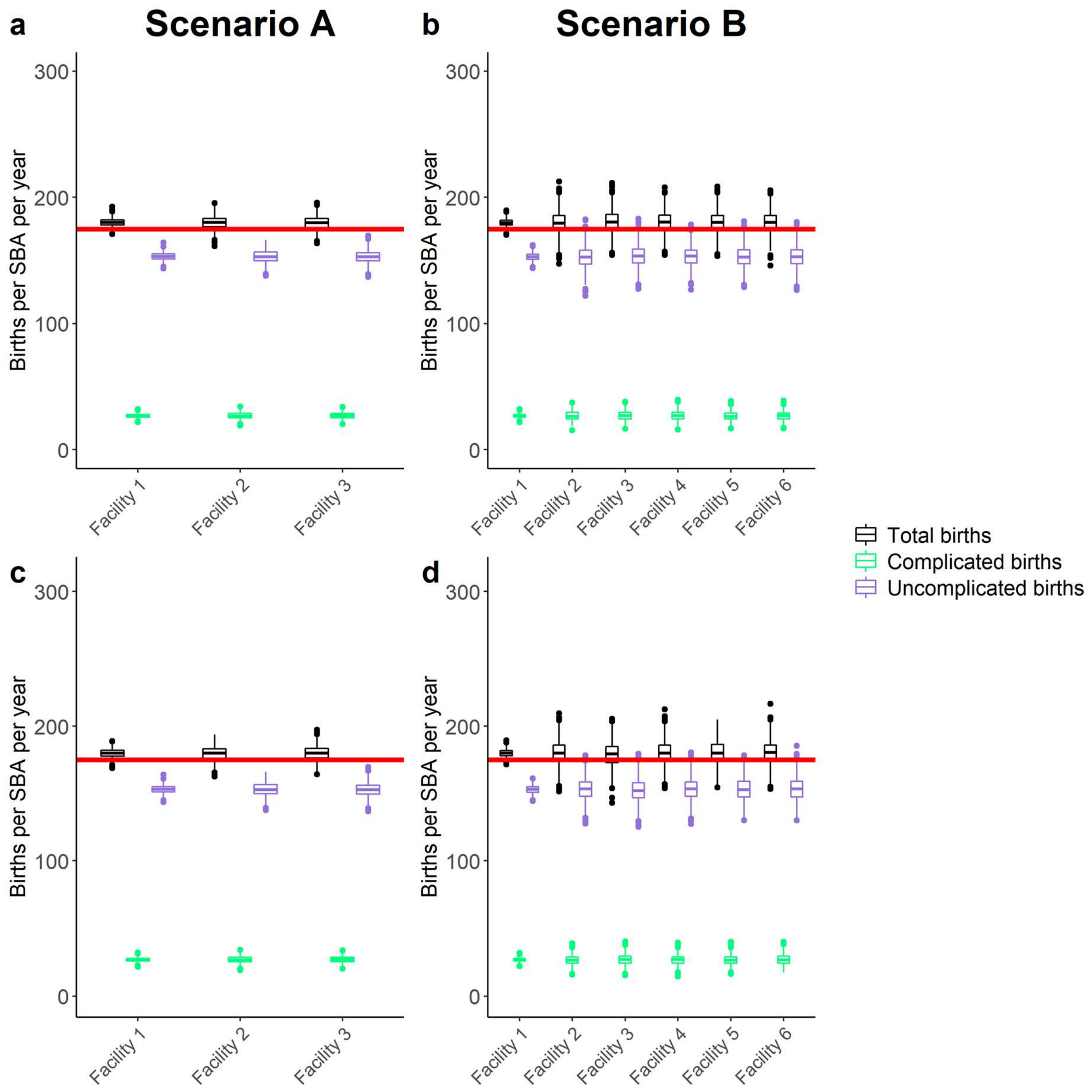

Figure S4: WHO Benchmark analysis. Distribution of uncomplicated and complicated births per SBA per year for each facility. Results from 1000 iterations of the stochastic simulation. Boxes: interquartile range. Whiskers extend to 1.5 times the interquartile range. Dots are outliers. The red line indicates 175 births pear year, the target number recommended by WHO.[1] WHO benchmark Scenario A: one CEmOC staffed by 10 SBAs and two BEmOCs staffed by five SBAs each; WHO benchmark Scenario B: one CEmOC staffed by 10 SBAs and five BEmOCs staffed by two SBAs each. Facility 1 in each scenario is a CEmOC. All other facilities are BEmOCs. BEmOC – Basic Emergency Obstetric Care Centre; CEmOC – Comprehensive Emergency Obstetric Care Centre; SBA – skilled birth attendant. Panels a and b: base case, assuming no birth seasonality. Panels c and d: results for a hypothetical setting with marked birth seasonality (see Methods for details).

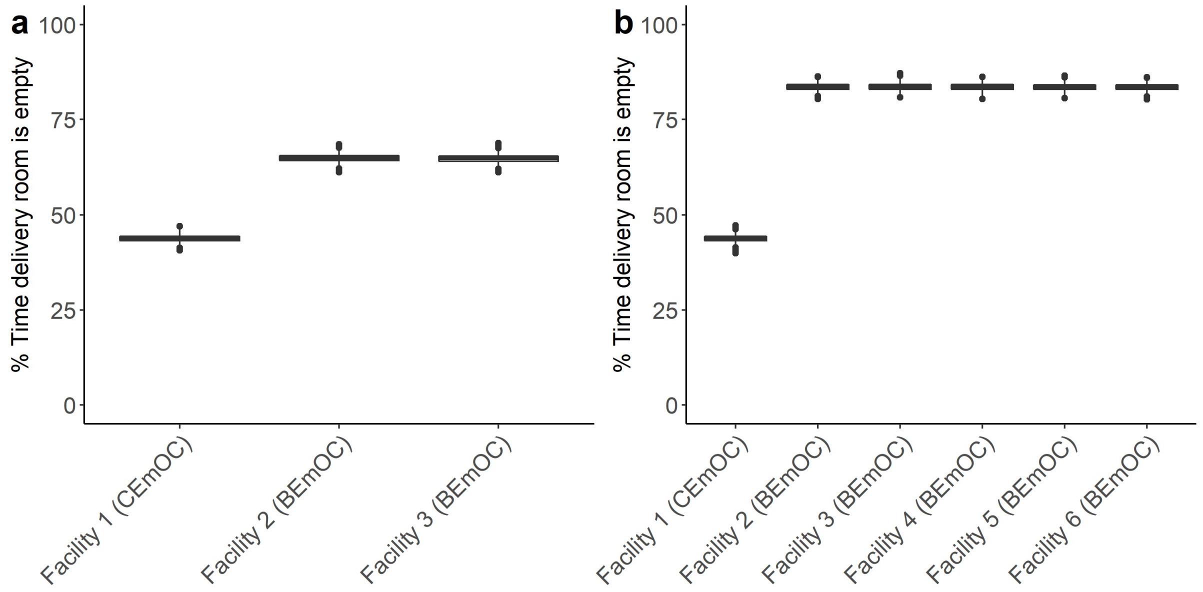

Figure S5: WHO Benchmark analysis with seasonality. Percentage of time over a year in which delivery rooms are empty. Results from 1000 iterations of the stochastic simulation. Boxes: interquartile range. Whiskers extend to 1.5 times the interquartile range. Dots are outliers. a) WHO benchmark scenario A: one CEmOC staffed by 10 SBAs and two BEmOCs staffed by five SBAs each; b) WHO benchmark scenario B: one CEmOC staffed by 10 SBAs and five BEmOCs staffed by two SBAs each. Facility 1 in each scenario is a CEmOC. All other facilities are BEmOCs. BEmOC – Basic Emergency Obstetric Care Centre; CEmOC – Comprehensive Emergency Obstetric Care Centre.

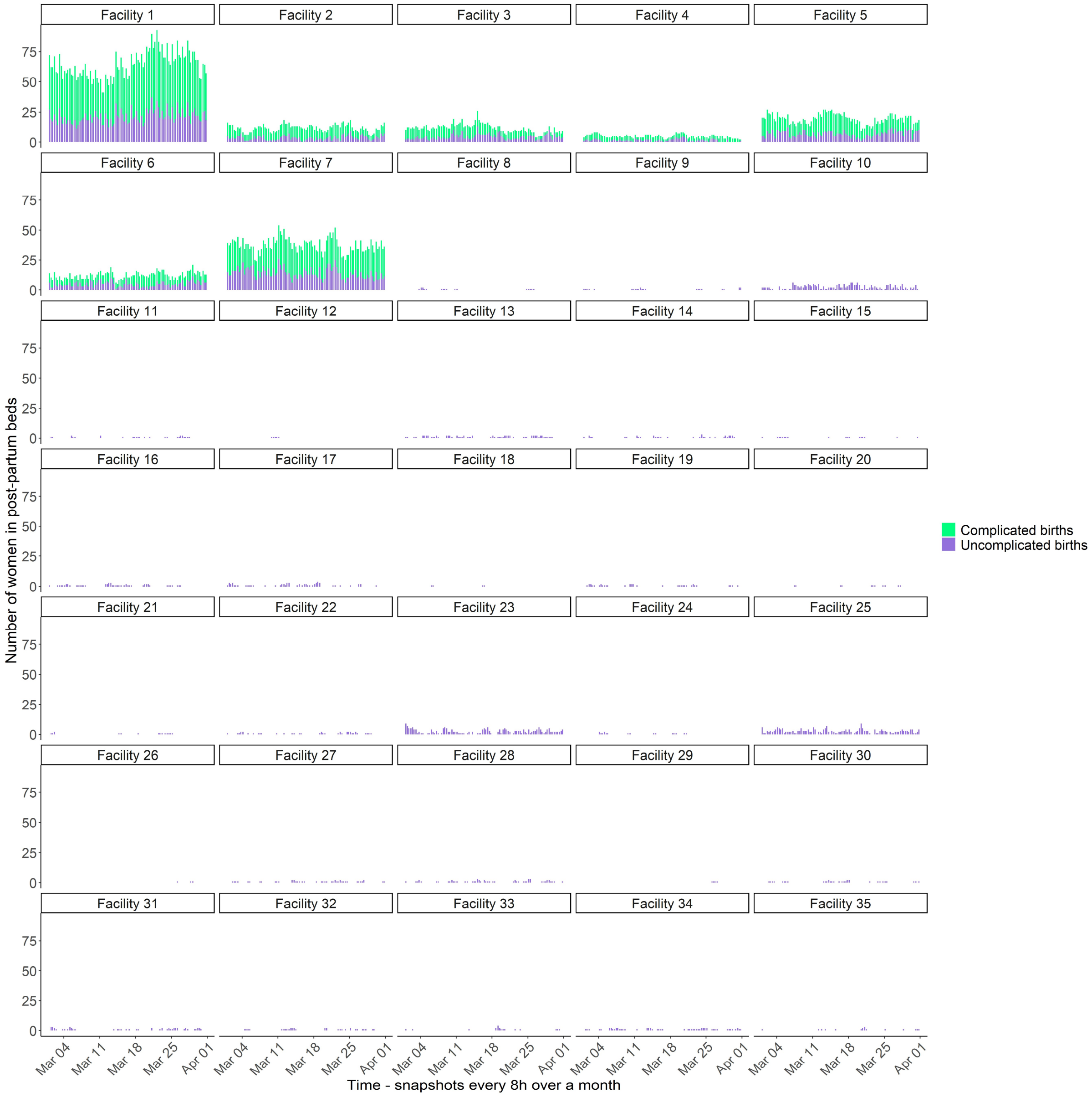

Figure S6: Zanzibar analysis. Simulated number of women in post-partum beds in 35 EmOC facilities in Zanzibar in 8h snapshots over a representative month. Purple bars indicate uncomplicated births. Green bars indicated complicated births. Seven facilities (Facilities 1 to 7) undertake Caesarean sections. Complicated births only occur in facilities that have been identified as CEmOCs and that are able to carry out Caesarean sections. Snapshots are spaced evenly. This means that if there are extended gaps between bars, these are shifts without any births in the facility.

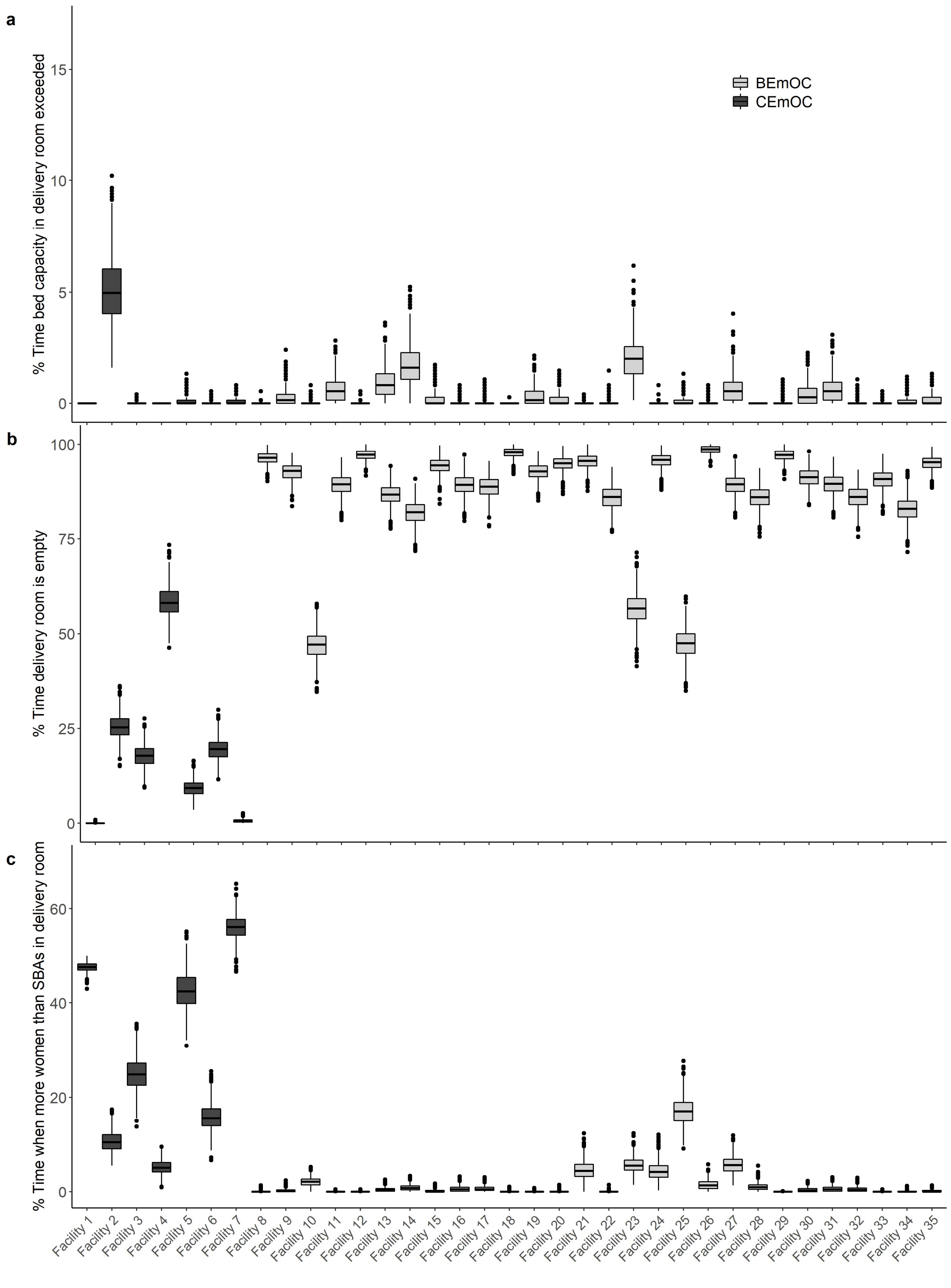

Figure S7: Zanzibar analysis. Occupancy statistics for 7 CEmOC facilities (Facilities 1-7) and 28 BEmOC facilities (Facilities 8-35) in Zanzibar. Results from 1000 iterations of the stochastic simulation. Boxes: interquartile range. Whiskers extend to 1.5 times the interquartile range. Dots are outliers. a) Percentage of time when bed capacity in delivery rooms is exceeded. b) Percentage of time when delivery rooms are empty. c) Percentage of time when more than four women per SBA are in a delivery room. BEmOC – Basic Emergency Obstetric Care Centre; CEmOC – Comprehensive Emergency Obstetric Care Centre; SBA – skilled birth attendant.

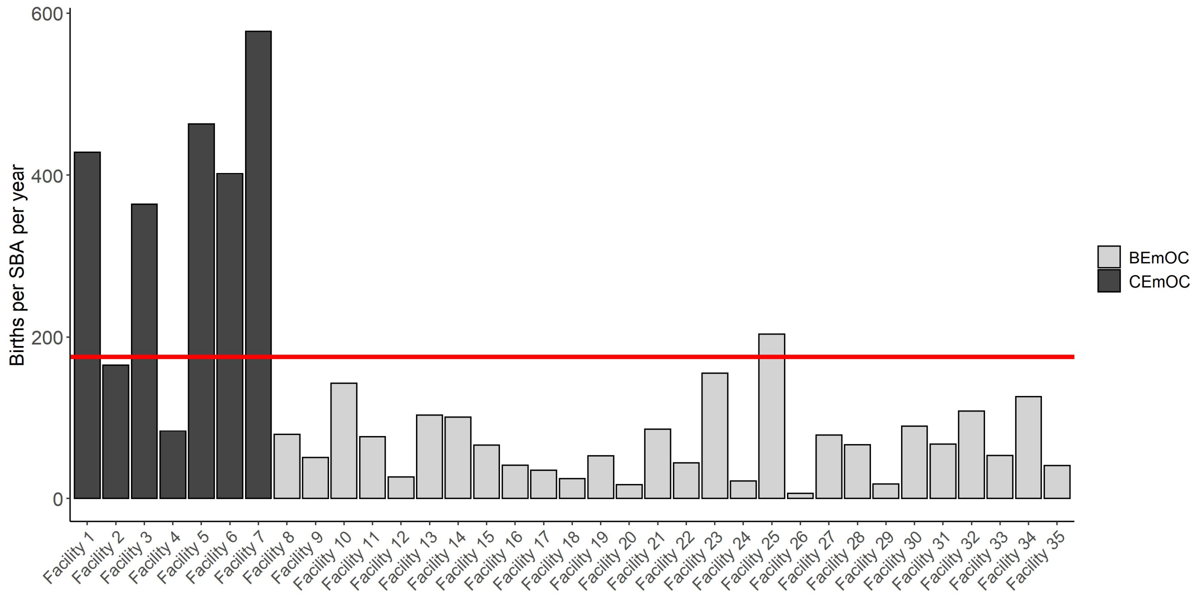

Figure S8: Zanzibar analysis. Expected number of births seen per SBA per year for 7 CEmOCs and 28 BEmOCs in Zanzibar. The red line indicates the 175 births per year recommended by WHO.[1] BEmOC – Basic Emergency Obstetric Care Centre; CEmOC – Comprehensive Emergency Obstetric Care Centre; SBA – skilled birth attendant.

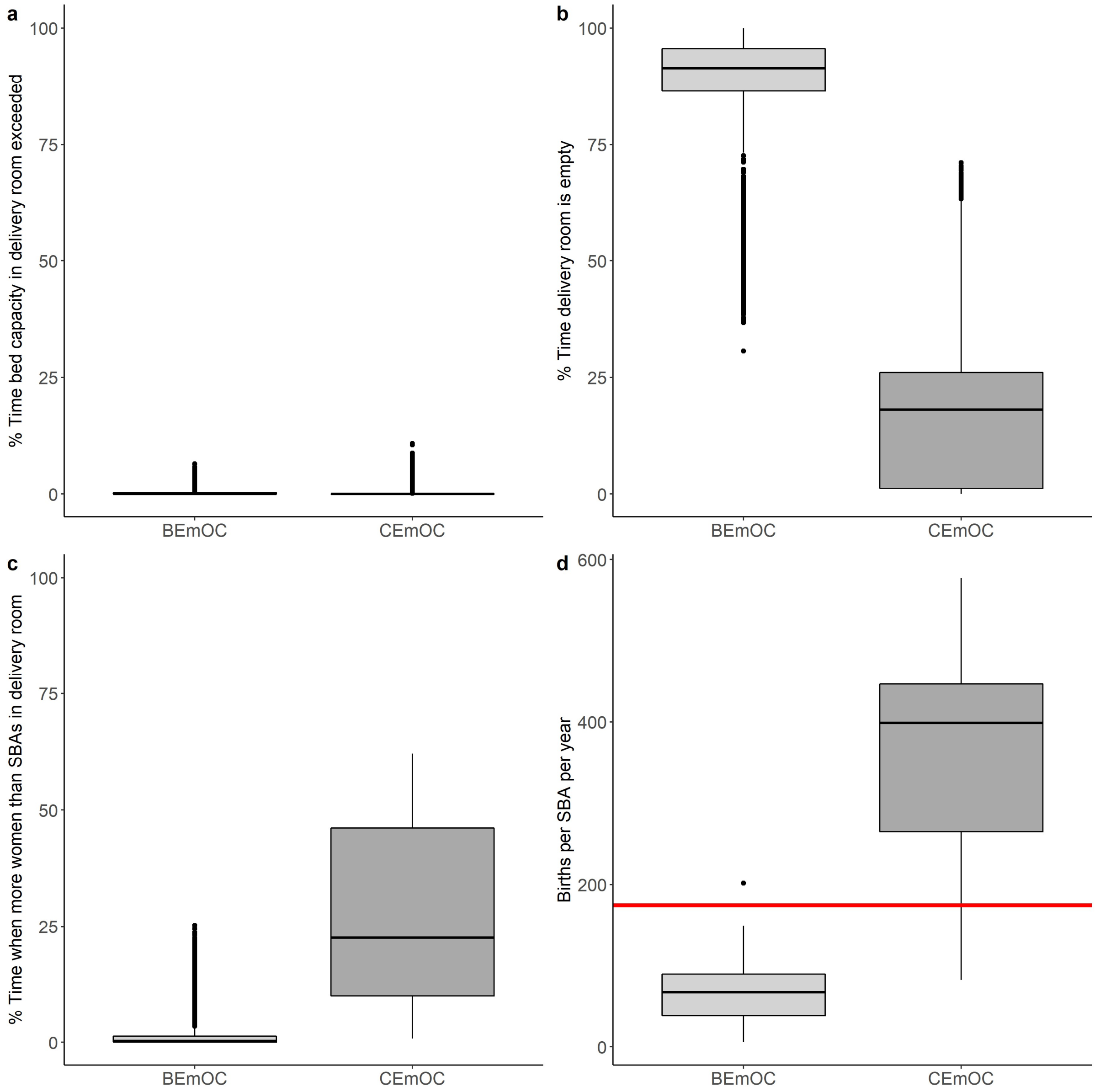

Figure S9: Zanzibar analysis with seasonality. Occupancy statistics for 7 CEmOC and 28 BEmOC facilities in Zanzibar. Results from 1000 iterations of the stochastic simulation. Boxes: interquartile range. Whiskers extend to 1.5 times the interquartile range. Dots are outliers. a) Percentage of time when bed capacity in delivery rooms is exceeded. b) Percentage of time when delivery rooms are empty. c) Percentage of time when more women than SBAs are in a delivery room. d) Births per SBA per year. The red line indicates the 175 births per year recommended by WHO. BEmOC – Basic Emergency Obstetric Care Centre; CEmOC – Comprehensive Emergency Obstetric Care Centre; SBA – skilled birth attendant.

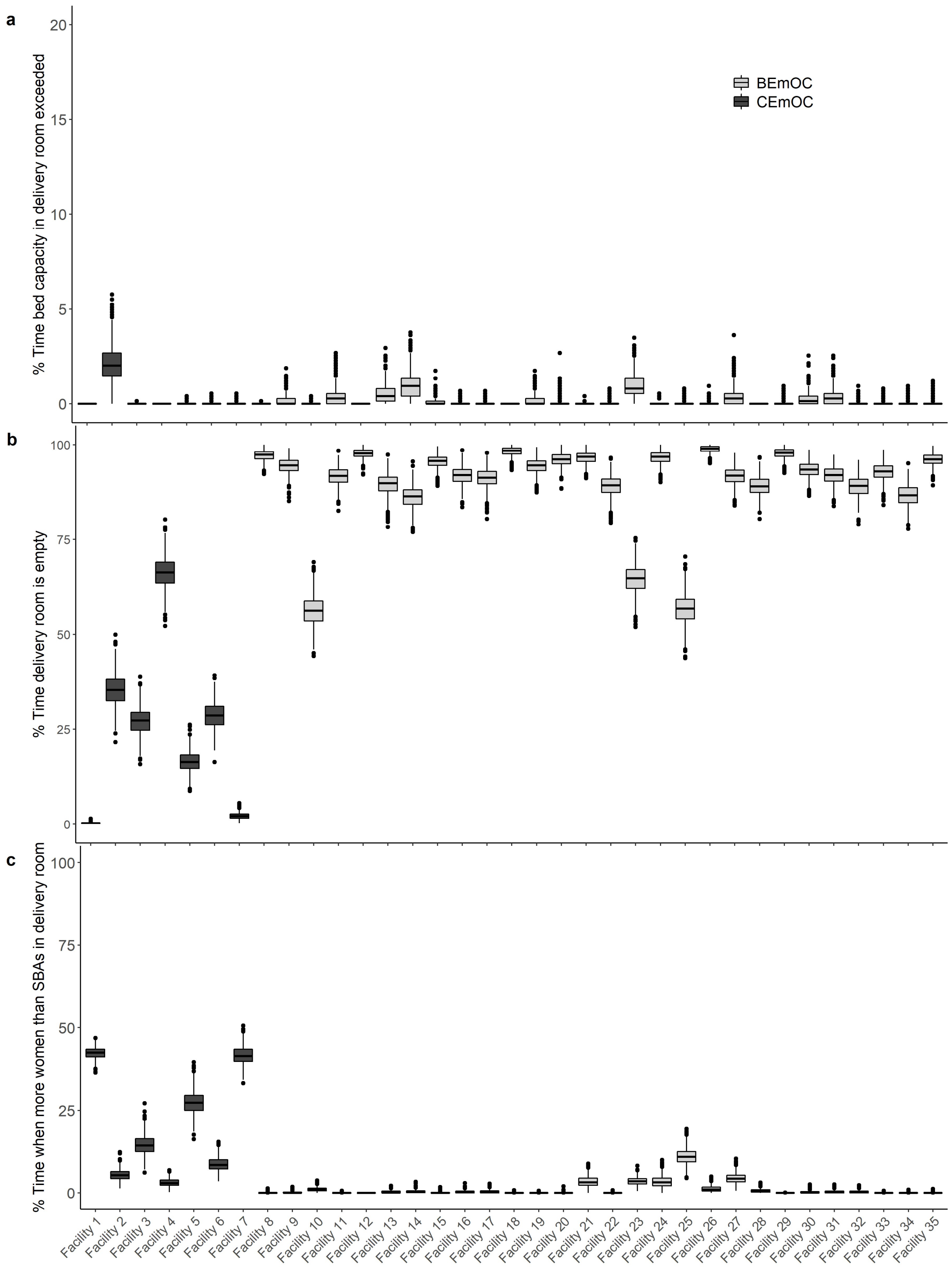

Figure S10: Zanzibar analysis with seasonality. Occupancy statistics for 7 CEmOC facilities (Facilities 1-7) and 28 BEmOC facilities (Facilities 8-35) in Zanzibar in the **least busy month (January)**. Results from 1000 iterations of the stochastic simulation. Boxes: interquartile range. Whiskers extend to 1.5 times the interquartile range. Dots are outliers. a) Percentage of time when bed capacity in delivery rooms is exceeded. b) Percentage of time when delivery rooms are empty. c) Percentage of time when more than four women per SBA are in a delivery room. BEmOC – Basic Emergency Obstetric Care Centre; CEmOC – Comprehensive Emergency Obstetric Care Centre; SBA – skilled birth attendant.

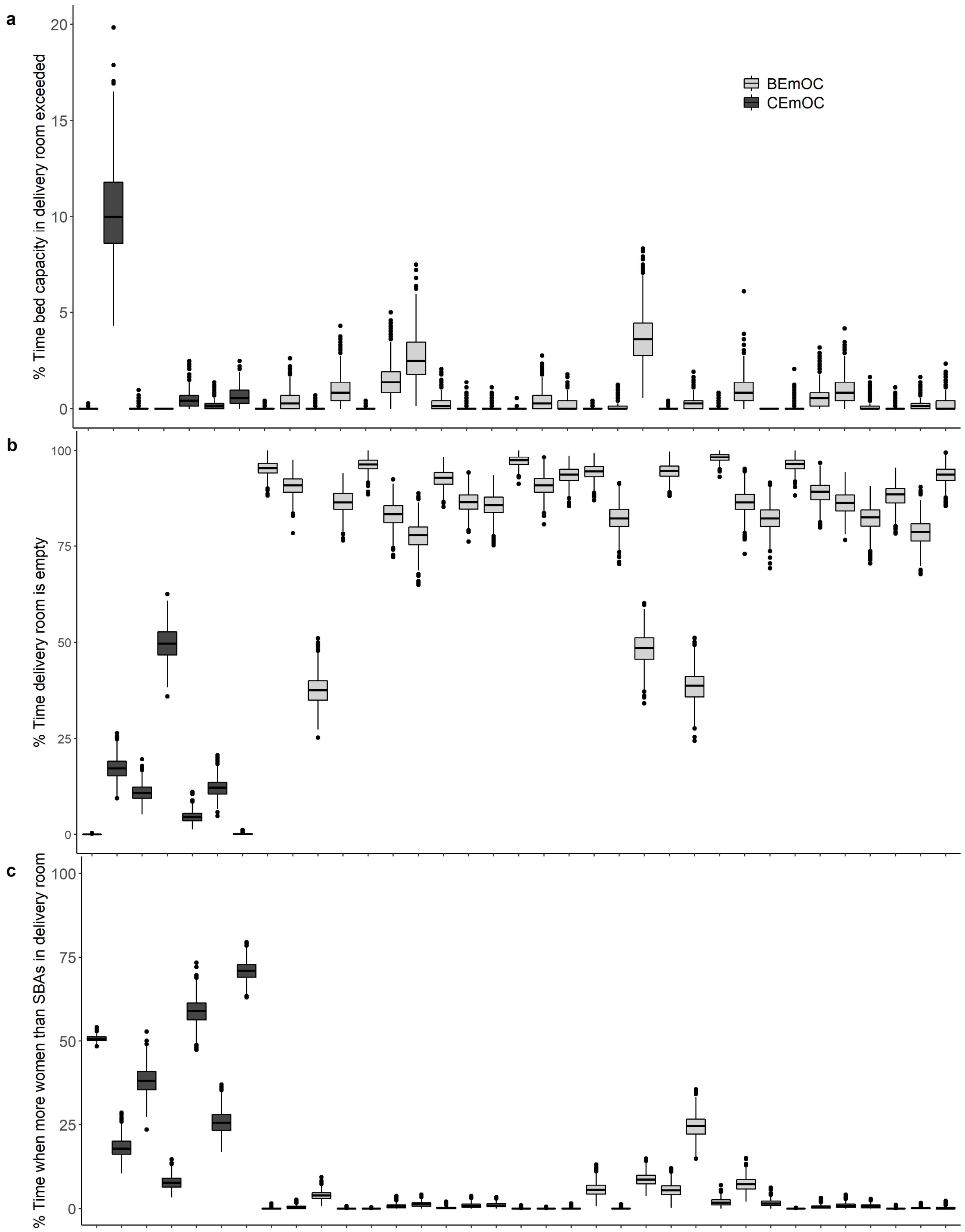

Figure S11: Zanzibar analysis with seasonality. Occupancy statistics for 7 CEmOC facilities (Facilities 1-7) and 28 BEmOC facilities (Facilities 8-35) in Zanzibar in the **busiest month (June)**. Results from 1000 iterations of the stochastic simulation. Boxes: interquartile range. Whiskers extend to 1.5 times the interquartile range. Dots are outliers. a) Percentage of time when bed capacity in delivery rooms is exceeded. b) Percentage of time when delivery rooms are empty. c) Percentage of time when more than four women per SBA are in a delivery room. BEmOC – Basic Emergency Obstetric Care Centre; CEmOC – Comprehensive Emergency Obstetric Care Centre; SBA – skilled birth attendant.

References

1. WHO. The World Health Report 2005. Make every mother and child count. Published 16th June 2005. Available from: https://www.who.int/publications/i/item/9241562900 Accessed 28th August 2022. 2005.
